# Revolutionizing dental research in the Pacific Islands: the Pacific Islands dental research framework

**DOI:** 10.3389/froh.2024.1464700

**Published:** 2025-01-07

**Authors:** Hemanth Tumkur Lakshmikantha, Ratu Osea Gavidi, Tokasa Leweni, Kantara Tiim, Kaitlyn Khan, Samantha Kumar

**Affiliations:** School of Dentistry and Oral Health, College of Medicine, Nursing and Health Sciences, Fiji National University, Suva, Fiji

**Keywords:** dental research, access to care, community, oral health disparities, patient-reported outcome measures (PROMs)

## Abstract

Traditional dental research paradigms often lack relevance in marginalized cultural contexts due to inherent biases and misalignment with local values. For Pacific Islanders, this issue is pronounced, as they face serious oral health challenges while remaining underrepresented in scientific discourse. In response, the authors developed the Pacific Islands Dental Research Framework (PIDRF), a culturally informed, community-driven model that directly addresses these limitations in conventional Western approaches. PIDRF supports indigenous priority-setting, reciprocal co-gauging, and cross-sector collaboration throughout the research process, guided by cultural relationship specialists and indigenous advisory boards. This framework expands diagnostic assessments to incorporate cultural and social determinants of oral health, combining holistic and culturally tailored therapies. By continuously integrating patient feedback, PIDRF fosters empathetic, effective support that aligns with Pacific values. PIDRF promotes knowledge-sharing, policy reform, and community-led advancements, enabling Pacific communities to lead in improving their oral health outcomes. This framework introduces an ethical, decolonized model for dental research, setting a new standard for culturally responsive research in Pacific contexts.

## Introduction

Dental research has seen substantive advances over recent decades—with evolutions in assessment tools, expanded insight into oral diseases, and new intervention techniques (1–3). However, most of the dental research has centered on Western contexts, norms, and frameworks (4–7). This has created significant knowledge and practice gaps regarding dental care needs, health outcomes, and research participation within diverse cultural contexts, including Pacific Island communities (8–10). Studies highlight that ethnic minority groups continue facing extensive dental health challenges but remain vastly underrepresented in research cohorts and agendas (11–13). For Pacific communities in particular, dental research often clashes with cultural values and worldviews tied to holistic well-being, community belonging, and participatory decision-making (6, 14–18). Mainstream methodologies rarely accommodate Pacific conceptions of health or respect the autonomy priorities of these marginalized populations. There is an increasingly urgent need for radical innovation in how dental research is conducted with and within Pacific populations. However, few existing frameworks effectively bridge gaps in cultural alignment and capacity to improve oral health access and equity for these communities.

Conventional dental research methodologies often fall short when applied in Pacific contexts due to inherent biases and assumptions carried over from Western frameworks. Core limitations include a lack of cultural contextualization of oral health constructs, inadequate participant engagement processes, and insufficient focus on positive psychology support (6, 17, 19). For example, major dental studies continue using clinical diagnoses and biomedical markers as primary measures of oral health status. However, for many Pacific communities, dimensions like social functioning, self-image, diet enjoyment, and community belonging bear equal or greater weight (20, 21). Additionally, research planning and interventions frequently proceed with minimal input from local populations and cultural leaders, hindering community ownership and sustainability (22–24). Even seemingly rigorous clinical trial designs tend to underestimate psychological anxiety related to dental procedures shaped by cultural identities and lived experiences (25). Methodologies rarely accommodate traditional healing practices, indigenous language incorporation, or holistic well-being correlates integral to Pacific conceptions of oral health. The resulting data and interventions struggle to generate meaningful improvements in access, equity, and outcomes. There is an extensive methodological realignment needed before dental research can produce translatable findings that elevate palliation and care for underserved Pacific communities.

In response to growing evidence of poor oral health outcomes and research mistrust among Pacific communities, various studies and reports have urgently appealed for radical innovation in dental research approaches for the region (26–28). There is consensus on the need to develop comprehensive frameworks specially tailored to the socio-cultural realities and expectations of Pacific populations to enhance engagement, relevance, and sustainability. This entails centering components like traditional healing practices, indigenous language incorporation, holistic well-being measures, and participatory decision structures. However, very few existing dental research frameworks effectively achieve meaningful cultural alignment, nuanced community participation, and localized capacity building (29–31). No dental studies in the Pacific over the past decade that fully integrated cultural advisors used indigenous taxonomies of oral health or implemented patient-centered adaptive designs and interventions. The gap remains cavernous regarding methodological models optimally bridging Western dental research norms with Pacific worldviews, priorities, and conceptions of wellbeing. There is both ample room and an urgent need for radical methodological innovation. Transformative new frameworks could set a gold standard for equity and empower Pacific communities to finally gain equitable footing and agency in the dental research realm.

The primary objective of this manuscript is to introduce and establish the Pacific Islands Dental Research Framework (PIDRF) as a new paradigm for dental research that specifically addresses the oral health needs of Pacific Island communities. Developed by the authors, PIDRF seeks to fill gaps in conventional Western research methodologies by prioritizing indigenous perspectives and fostering participatory approaches. This framework aims to provide culturally informed and community-led solutions that align with the unique values and health priorities of Pacific Island populations, promoting both research relevance and long-term community impact.

## Pacific Islands dental research framework's (PIDRF)

**Figure 1:**
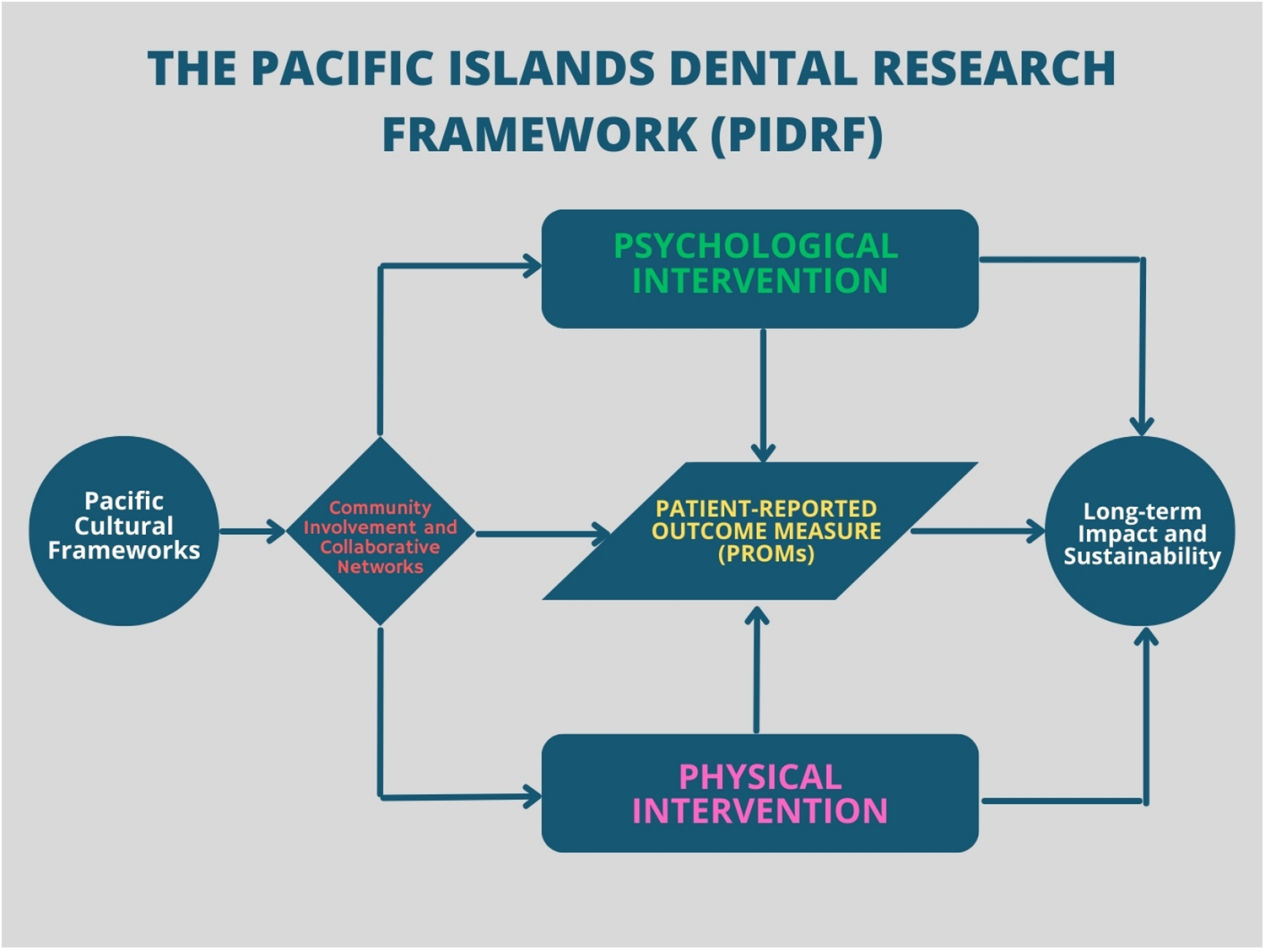
The Pacific Islands Dental Research Framework (PIDRF) illustrates the interconnected components essential for dental research in the Pacific context. The framework begins with Pacific Cultural Frameworks, which inform Community Involvement and Collaborative Networks. These networks influence both Psychological and Physical Interventions, which converge on Patient-Reported Outcome Measures (PROMs). The framework culminates in Long-term Impact and Sustainability, emphasizing the cyclical nature of sustainable dental research in Pacific Island communities.

At its foundation, PIDRF emphasizes participatory, decolonized, culturally grounded inquiry as the guidepost for research conceptualization and planning (Figure 1). This is evidenced methodologically through four major principles, namely, (1) Community-First Co-Design, (2) Biocultural Ethics Integration (3) Holistic Wellbeing Indexes, and (4) Sustainable Systems Thinking. To uphold these principles, PIDRF relies on four central components: (A) Cultural Bridging Consultants, (B) Indigenous Advisory Councils, (C) Multimodal Intervention Packages, and (D) Patient-Reported Feedback Loops. The Cultural Bridging Consultants ground community involvement based on local conventions, interactions, and collaborations to clarify the agendas and relevant participation throughout the phases. Indigenous Advisory Councils complement researcher teams to provide cultural perspectives on variables and measures, analysis and interpretation. Multimodal Intervention strategies allow for customized physical-psycho-social support plans for communities. Finally, Patient Reported feedback Loops involves acquiring participant's opinions regarding the study hence facilitating utilization of feedback for further tuning in the effort to achieve the best aligned and influential frameworks. Altogether PIDRF aspires to be a new paradigm for oral health research within and alongside Pacific communities.

PIDRF's recruitment processes rely deeply on Cultural Bridging Consultants to determine appropriate cultural protocols, community leaders to liaise with, indigenous language adaptations needed in study information, and participatory consent models aligning research with community values. This establishes early cultural grounding and shared agency in the research process for participants. The framework moves beyond clinical dental measures to gather holistic self-reported and observational data linking oral health to spiritual, communal, emotional, physical, and cultural well-being from participant perspectives. Quantitative and qualitative instruments are collaboratively designed under the guidance of Indigenous Advisory Councils incorporating relevant cultural health indicators. Interventions fuse evidence-based clinical dental treatments with traditional healing practices and folk therapies as defined by a joint committee of practitioners, participants, and Bridging Consultants. The customized regimens address biological factors alongside social, psycho-emotional, and spiritual dimensions of oral health unique to Pacific communities. Structured participatory dialogues, surveys, and journals systematically capture patient reflections on their interventions—including alignment with cultural values, the efficacy of blended care modalities, impacts on identity and belonging, and lived functionality related to oral health or illness. Such data allows for progressive refinement of interventions and measurements.

### A. Cultural alignment strategies

This fundamental aspect entails the framework's approaches to prioritize indigenous wisdom and integrate community feedback through all stages (30–32). Key aspects include:

#### Centering of indigenous wisdom

• Incorporation of traditional healing practices: PIDRF allows for practices of the tribal healers; including medicinal plants, application of herbal compresses, and folk remedies for oral diseases with traditional knowledge passed across generations can be integrated.
• Integration of native disease taxonomies: The methodology integrates Pacific languages’ unique names, symptoms, and causes of dental diseases bounded in ancestral experiences—avoiding biased Western disease labels and definitions.
• Drawing on the wisdom of past generations: Planning dialogues involve integrating the teachings of ancestors. These teachings shape the values of the community, emphasizing sustenance, prevention, living in harmony with the environment, and collective well-being. This approach ensures that dental research reflects the community's perspectives and beliefs.

#### Community participation approaches

• Co-design of research questions and variables: Community discussions (Talanoa) inform the selection of relevant dental health variables resonating locally while villagers shape research topics most impacting access and equity.
• Collaboration on consent processes and data-gathering techniques: Cultural consultants provide guidance on ethical adaptations to study information and consent materials while co-developing research instruments capturing dental health influences within a cultural lens.
• Joint development of targeted interventions: Combining scientific and traditional healers’ expertise, multi-modal interventions incorporate tribal practices for dental related issues, psycho-social dimensions of dental illness, and promoting holistic wellness.
• Ongoing feedback loops on framework effectiveness: Continual feedback, captured using cultural communication norms, enables iterative improvements of interventions that best align with community priorities and dental health conceptions.

The combined cultural grounding philosophies and community partnership structures allow PIDRF to meaningfully bridge indigenous worldviews with research through active participation, power sharing, and validating marginalized Pacific knowledge systems. This upholding of biocultural ethics provides the scaffolding for relevant dental research.

### B. Community involvement and collaborative networks

A cornerstone of PIDRF's participatory ethos involves formally integrating both grassroots and institutional input across the dental research process through diverse community involvement and collaborative multisector networks (33). At the grassroots level, Pacific community members, cultural experts, traditional healers, and village leaders help shape research priorities, variables, designs, and interventions via standing advisory councils that hold decision-making authority. Simultaneously, national health agencies, dental associations, NGOs, and academic institutions align to provide specialized personnel, advisory support, advocacy platforms, funding mechanisms, and policy development frameworks to accelerate adoption, learning, and scale-up initiatives. This interplay of bottom-up and top-down collaboration allows PIDRF to synthesize participatory community insights while leveraging establishment resources for maximum impact and sustainability. The framework's emphasis on equitable, decolonized partnerships between Pacific peoples and supporting external institutions sets a new standard for community-anchored capacity building and mutual advancement.

### C. Holistic health assessments

Unlike conventional methodologies, PIDRF has been purposely designed to include systematic integration of Patient-Reported Outcome Measures (PROMs) to improve the specificity of interventions and to understand overall impacts in the participants’ own words (34, 35). This includes regular documentation of patients lived experiences, cultural sensitivity, as well as their views on the changes in dental health, self-image, activity levels and psycho-social well-being. Collection is based on culturally safe tools co-created with the community councils, which include storytelling and check-ins based on the community's metaphors of health and journals. Evaluation incorporates native perceptions of changes in health with reference to the community's concerns such as satisfaction with food intake that is traditional and reduced pain as it hampers certain cultural practices. Patient-derived indices enable a shift back to patient-centered approaches to identify the most readily palatable forms of intervention while at the same time educating patients about how better oral health is interconnected with Pacific views on health and functioning. PIDRF maintains the PROM-centered assessment fabricating research accountability to patients and unearthing the community's strength to develop designs offering optimal relevance.

### D. Patient-reported outcome measures (PROMs)

In contrast to a conventional operational procedure, PIDRF proactively and consistently applies PROM correctly for adaptive adjustments in the interventions, as well as to assess the structures’ broader impressions from the participants’ standpoint (36–41). Data collecting uses decolonized tools created in concert with community councils that incorporate storytelling, check-ins utilizing local metaphors of health and journaling. Analysis welcomes indigenous conceptions of health that fit local concerns such as nutrition satisfaction and reduced pain influencing community rituals. Patient-defined measures support participatory learning on how better oral health interacts with Pacific concepts of wellness and functionality while allowing recalibration towards ideally resonant treatments. Maintaining PROM-centered assessment helps PIDRF to empower community voices to build designs with greatest relevance and improve research responsibility to patients.

### F. Long-term impact and sustainability

To ensure the long-term impact of the Pacific Islands Dental Research Framework (PIDRF), securing sustainable funding and resources is critical. Given PIDRF's community-centered and culturally aligned approach, partnerships with governmental, non-governmental, and international organizations will be essential for both initial and ongoing support. These partnerships can provide critical funding channels, advocacy for policy changes, and resources for capacity building among local researchers and healthcare providers.

For enduring sustainability, PIDRF also emphasizes the development of local expertise and infrastructure within Pacific communities. This includes training local healthcare workers, establishing community health programs, and advocating for policies that support traditional healers and indigenous health practices. By investing in workforce development and building local capacity, PIDRF seeks to reduce dependency on external resources and ensure that Pacific communities have the skills and support needed to sustain their dental health initiatives independently. Additionally, the framework promotes participatory education programs and fosters leadership within communities to advocate for continued resources and policy support, ultimately creating a self-sustaining model for community-led oral health improvements.

## Implementation considerations

Fulfilling PIDRF's transforming ambition calls for overcoming several resources, capability, and adoption constraints preventing actual research progress. While overcoming institutional resistance, effective implementation depends on, (1) adequately provisioning multi-sector partnerships, participatory structures, and specialized skill sets while (2) overcoming systemic inertia.

### Resource needs and partnership dynamics

To support the clear community co-design features, the framework calls for significant time, money, staff, and governance partnerships investments. Lead researchers must gather community representatives and assign staff members to participate in cultural sensitivity reviews and participatory analysis, therefore necessitating institutional commitment. At scale, practical implementation frameworks depend on government health agencies and dental associations offering specialized knowledge, data infrastructure, and policy inputs. Negotiating these challenging relationships improves translational capacity, research integrity, and output sustainability while creating administrative challenges.

### Overcoming adoption barriers

Changing traditional dental research systems anchored on conventional clinically governed, intervention-oriented approaches call for facing systemic rigidity. PIDRF's heightened expectations on cultural fluency, qualitative techniques, community-driven goals, and traditional healing limit familiarity and call for new quality assessment methodologies. Moreover, undervaluation of resource-intensive cooperation inherent in PIDRF results from prevailing incentive systems. Adoption can be driven by strategic consensus-building with gatekeeping institutions around supportive funding sources, interactive training programs, and benefit demonstrations.

### Addressing practical implementation challenges

Implementing the Pacific Islands Dental Research Framework (PIDRF) across diverse Pacific Island communities presents several practical challenges. One primary obstacle is the need for researcher adaptability and cultural fluency, as the framework requires sensitivity to varying cultural norms, languages, and community structures. Additionally, engaging with traditional healers and local leaders may introduce variability in research processes, as perspectives can differ significantly across regions. Another challenge involves aligning community expectations with research objectives, especially when integrating traditional practices with evidence-based dental treatments. To address these complexities, PIDRF emphasizes the importance of cultural bridging consultants and participatory consent processes to establish mutual understanding and co-governance from the outset. Finally, long-term sustainability is critical yet challenging, as maintaining the framework's participatory approach requires adequate funding and policy support. By fostering partnerships with local governments, NGOs, and health organizations, PIDRF aims to create a foundation for sustainable research practices that can thrive beyond initial project phases.

Validation of the Pacific Island Dental Research Framework (PIDRF) is a critical next step to ensure its applicability and effectiveness across diverse Pacific Island communities. While PIDRF is currently presented as a conceptual framework grounded in cultural, participatory, and ethical principles, plans are underway to implement pilot studies that will empirically test its components. These studies aim to evaluate the framework's feasibility, cultural alignment, and its ability to improve oral health outcomes through integrated psychological, physical, and community-based interventions. Data collected from these pilot initiatives will refine the methodology, allowing for iterative development and stronger alignment with participant needs and regional oral health challenges. This empirical validation will serve as the foundation for broader implementation, ensuring the model's adaptability and effectiveness in addressing oral health inequities.

## Impact and implications

PIDRF establishes paradigm-shifting parameters globally for decolonizing dental research and presents a possible breakthrough for improving access, self-determined participation, and oral health equity among underserved Pacific populations.

### The transformative potential for pacific communities

By directly addressing cultural estrangement factors that have marginalized Pacific peoples, PIDRF offers a gateway for restored agency, dignity, and visibility within dental realms regionally and abroad. The framework promises to dramatically elevate community trust, engagement, funding representation, and collective capacity to reclaim the direction of oral health outcomes locally. Shared authority and participatory structures also promise psychological validation while advancing traditional knowledge.

### Anticipated concerns and alternative perspectives

While the Pacific Islands Dental Research Framework (PIDRF) offers an innovative, culturally grounded approach to dental research, it also acknowledges potential concerns and alternative perspectives. One anticipated critique may focus on the contrast between indigenous and Western research methodologies, particularly regarding the integration of traditional healing practices alongside scientific rigor. To address this, PIDRF incorporates indigenous advisory councils and cultural bridging consultants, who help guide the research process and uphold both methodological integrity and cultural relevance. Additionally, there may be concerns about the logistical complexities and time requirements for implementing such a community-centered model. Recognizing these challenges, PIDRF promotes adaptable frameworks that allow research teams to adjust methods according to local customs and resources. This flexibility, combined with regular community feedback loops, enables the framework to maintain alignment with participant expectations and values, which is essential for fostering trust and engagement in Pacific Island communities.

### Setting new global standards

PIDRF signifies a breakthrough model for how to conduct ethical, empowering, and clinically impactful dental research among vulnerable populations everywhere. The foundational challenge to individualistic, biomedically oriented research doctrine stands to reshape standards worldwide, accelerating progress towards health equity. PIDRF philosophies and practices around decolonized methodologies, participatory priority-setting, cultural pluralism in interventions, community-led analysis, and collective ownership over outputs carry universal relevance to elevating research integrity everywhere. PIDRF's international demonstration effect can spur rapid transformation.

## Conclusion

PIDRF is a novel dental research technique developed especially to meet underserved oral health needs in underprivileged Pacific Island communities. PIDRF provides a set of innovative perspectives to essentially adapt dental research activities to the socio-cultural priorities, values, and concepts of health maintained by Indigenous Pacific people. Deep community co-governance and priority-setting; comprehensive biopsychosocial assessments; patient-reported feedback systems; capacitating Pacific workforces for self-determined, locally sustained leadership in oral health; core framework tactics with their fundamentally decolonizing roots, PIDRF upholds ethical obligations and drives more powerful, reliable, and easily available treatments projected to redress injustices confronting underprivileged Pacific populations. Furthermore, PIDRF's ideas on participatory, culturally focused research offer a paradigm with worldwide consequences for similarly empowering underprivileged populations outside of the Pacific. For our increasingly varied society, PIDRF represents a research paradigm change driving more democratic, pluralistic, fair, and responsive dentistry investigation.

## Data Availability

The raw data supporting the conclusions of this article will be made available by the authors, without undue reservation.
